# Opportunistic parasitoses among Egyptian hemodialysis patients in relation to CD4+ T-cell counts: a comparative study

**DOI:** 10.1186/s12879-019-4110-4

**Published:** 2019-05-29

**Authors:** Amany I. Shehata, Faika Hassanein, Rashad Abdul-Ghani

**Affiliations:** 1 Department of Tropical Health, High Institute of Public Health, Alexandria University, Alexandria, Egypt; 2 Department of Microbiology and Immunology, Faculty of Pharmacy and Drug Manufacturing, Pharos University, Alexandria, Egypt; 3 Department of Medical Parasitology, Faculty of Medicine and Health Sciences, Sana’a University, Sana’a, Yemen; 4 Tropical Disease Research Center, Faculty of Medicine and Health Sciences, University of Science and Technology, Sana’a, Yemen

**Keywords:** Intestinal parasitosis, *Toxoplasma gondii*, Hemodialysis, CD4+ Tcell, Egypt

## Abstract

**Background:**

Some reports are available on the prevalence of opportunistic parasitoses among hemodialysis (HD) patients, yet there is a paucity of data on the association of CD4+ T-cell counts with such infections. Therefore, this study aimed to determine the prevalence of intestinal parasites and *Toxoplasma gondii* in relation to CD4+ counts among HD patients in Alexandria, Egypt.

**Methods:**

A comparative cross-sectional study was conducted on 120 HD patients and 100 apparently healthy individuals between December 2014 and January 2016. Data and samples (stool and blood) were collected from the participants after obtaining their informed consent. Stool samples were examined for parasites after concentration and staining, EDTA-blood samples were used for CD4+ counting by flow cytometry, and sera were analyzed for anti-*Toxoplasma* IgM and IgG antibodies.

**Results:**

A significantly higher prevalence rate of intestinal parasitoses was found among HD patients compared to apparently healthy individuals (52.5% vs. 12.0%, respectively), with absence of helminths. *Cryptosporidium* species (32.5%), *B. hominis*(24.2%) and microsporidia (11.7%) were the most frequent parasites among HD patients, while *B. hominis* (13.0%), *Cryptosporidium* species (11.0%) and *G. lamblia* (4.0%) were the most frequent parasites among their counterparts. Statistically significant differences in parasite infection rates between patients and their counterparts were found for *Cryptosporidium* species, *B. hominis* and microsporidia. However, parasite species were not significantly associated with diarrhea. On the other hand, the overall *T. gondii* seroprevalence rate among HD patients was significantly higher than that among their counterparts (33.3% vs. 8%, respectively). HD patients with CD4+ counts < 200 cells/μl were twice more exposed to intestinal parasitoses compared to those with counts ≥200 cells/μl, but the difference was not statistically significant. However, low CD4+ counts were significantly associated with higher rates of *Cryptosporidium* species, microsporidia and *T. gondii*.

**Conclusions:**

Intestinal parasitoses and *T. gondii* infection rates are significantly higher among Egyptian HD patients compared to apparently healthy individuals, with *Cryptosporidium* species, *B. hominis*, microsporidia and *T. gondii* being the most frequent parasites. CD4+ counts < 200 cells/μl are significantly associated with *Cryptosporidium* species, microsporidia and *T. gondii* among HD patients. Therefore, regular screening of HD patients for opportunistic parasites is recommended.

## Background

Renal failure is an immunosuppressive condition that makes patients more prone to infections, including those caused by opportunistic protozoan parasites. Patients undergoing hemodialysis (HD) suffer from humoral and cell-mediated immune defects and have disturbances in acquired immune response to a variety of antigens [1, 2]. End-stage renal failure (ESRF) leads to impaired cell-mediated immunity as a result of lymphopenia and dysfunction of cluster of differentiation 4 (CD4+) T cells [1]. Moreover, uremia-associatedpro-inflammatory conditions developed in ESRD patients can lead to irreversible premature aging of T-cell compartment that contributes to the cell-mediated defects and susceptibility to infectious agents [3, 4]. On the other hand, HD induces apoptosis of T cells, decreased phagocytic capacity of neutrophils and monocytes along with abnormal production of pro-inflammatory cytokines by monocytes because of their direct contact with artificial membranes in HD machines [5–8]. In addition, frequent blood transfusions to patients with chronic renal failure (CRF) can lead to the development of antibodies against human leukocyte antigens, which can form immune complexes that interfere with cellular immune responses [9, 10].

Opportunistic parasitic infections, including those with *Cryptosporidium* species*, Cystoisospora belli, Cyclospora cayetanensis, Blastocystis hominis, Toxoplasma gondii* and microsporidia, have been documented to cause serious complications or even death among immunocompromized patients, including those undergoing HD [11–13]. In this context, humoral and cell-mediated immunosuppression can increase the susceptibility to and worsen the outcome of parasitic infections through increasing the acquisition of infection and the clinical severity of disease [14].

In Egypt, ESRD has been estimated to affect 264 to 414 per million population [15]. Despite the availability of a number of reports on opportunistic parasitoses among HD patients in Egypt [16–18], there is a paucity of data on the association of CD4+ counts with such infections. Therefore, the present study was conducted to determine the prevalence of intestinal parasites and *T. gondii* among HD patients in Alexandria governorate, Egypt in relation to CD4+ counts compared to apparently healthy individuals.

## Methods

### Study design, subjects and ethical considerations

A comparative cross-sectional study was conducted on 120 HD patients from different hospitals and 100 apparently healthy individuals in Alexandria, Egypt from December 2014 to January 2016. According to hospital records, patients were screened for human immunodeficiency virus (HIV) on admission and before the start of HD sessions, and those HIV-positive were referred to special hospitals. HD patients included in the study had no other immunosuppressive conditions, were not undergoing an immunosuppressive therapy and had not received antiparastitic treatment the month preceding the study. On the other hand, apparently healthy individuals of matched age, sex and residence were included provided that they had no kidney problems or immunosuppressive conditions, were not undergoing an immunosuppressive therapy and had not received antiparasitic treatment the month preceding the study.

### Data and sample collection

Data on the gender, age, residence, education, occupation, presentation with diarrhea and CD4+ count of study participants were collected using a pre-designed questionnaire after obtaining their informed consent to voluntarily participate in the study. Fresh stool samples were collected into clean, pre-labelled containers on two successive sessions for HD patients, where all patients were subjected to three sessions a week, and on two alternate days for apparently healthy individuals. Patients were classified as diarrheic if they had reported the passage of loose or liquid stools three times or more per day or if they were suffering from passing more frequent loose bowel movements than normal each day [19], either on the day of sample collection or in the 2 weeks prior to sample collection.

Approximately 5 ml of venous blood was collected from the patients on the day of the collection of first stool samples by nurses of the centers for patients and by one of the researchers for the apparently healthy individuals. The collected blood was divided equally (2.5 ml each) into a plain test tube and an EDTA-tube. The blood in plain tubes was left to clot, and the sera were then separated by centrifugation at 3000 rounds per minute for 5 min., dispensed into pre-labelled Eppendorf tubes and stored at − 20 °C until the detection of anti-*Toxoplasma* antibodies. The EDTA-anticoagulated blood was used for counting CD4+ cells. Stool and sera were transferred to the Parasitology Laboratory of the High Institute of Public Health, Alexandria University for processing and examination. However, EDTA-blood samples were transferred within a maximum of 2 h to the Hematology Laboratory of the Clinical Pathology Department, Faculty of Medicine, Alexandria University for CD4+ cell counting.

### Parasitological investigations

Two permanently stained smears were prepared from fresh stool samples and then stained with trichrome and quick-hotGram-chromotrope techniques for the detection of protozoan trophozoites and microsporidian spores, respectively [20–22]. The remaining stool samples were concentrated by formalin-ethyl acetate sedimentation concentration technique and examined for protozoan cysts and helminthic ova [23]. A smear was then prepared from the sediment and stained by the modified Ziehl-Neelsen technique for detecting coccidian oocysts [24, 25]. The identification and differentiation of intestinal parasites were based on the diagnostic criteria and bench aids provided by the World Health Organization [26].

### CD4+ T-cell counting

EDTA-blood samples were immediately processed for counting CD4+ cells according to standard procedures [27], using the Attune™ NxT Flow Cytometer (Thermo Fisher Scientific, Waltham, MA, USA). Because CD4+ counts show variability in the absence of HIV infection [28], we used a threshold level of 200 CD4+ count/μl to study the association of low CD4+ count with parasitoses because the counts below 200/μl is considered the severe level of immunosuppression that can be associated with opportunistic infection [29].

### Detection of anti-*Toxoplasma* antibodies

Anti-*Toxoplasma* IgM and IgG antibodies were detected in sera using enzyme-linked immunosorbent assay kits (BioCheck Inc., South San Francisco, CA, USA) according to the manufacturer’s instructions. The cut-off points for IgM and IgG seropositivity were 1 IU/ml and 32 IU/ml, respectively, where no sera showed borderline reactivity.

### Statistical analysis

Data were entered, edited and analyzed using IBM SPSS Statistics, version 20.0 (IBM Corp., Armonk, NY, USA). Categorical variables were presented as frequencies and percentages and compared using chi-square test or Fisher’s exact test, whichever suitable, while continuous variables were presented as means with standard deviations and compared using *t*-test. Differences and associations were considered statistically significant at *P* values < 0.05.

## Results

### Characteristics of the study subjects

Table 1 shows the detailed characteristics of HD patients and the comparison group. The mean age and CD4+ count of HD patients was 50 ± 13.41 years (range: 20–80) and 934 ± 124 cells/μl, respectively. About a third of the HD patients presented with diarrhea (30%; 36/120) and 17.5% (21/120) had CD4+ counts of < 200 cells/μl. On the other hand, the mean age and CD4+ count of the apparently healthy individuals were 48.92 ± 16.32 years (range: 20–80) and 3490 ± 2761 cells/μl, respectively. In addition, all apparently healthy individuals had CD4+ counts of > 500 cells/μl and only one patient (1.0%) presented with diarrhea (Table 1).

**Table 1:** Characteristics of HD patients and their apparently healthy counterparts in Alexandria (2014–2016)

Characteristic	HD patients (*N* = 120)	Apparently healthy individuals (*N* = 100)
	*n* (%)	*n* (%)
Gender		
Male	58 (48.3)	47 (47.0)
Female	62 (51.7)	53 (53.0)
Age (years)		
Mean ± SD	50.21 ± 13.41	48.92 ± 16.32
20–39	31 (25.8)	31 (31.0)
40–59	54 (45.0)	39 (39.0)
≥ 60	35 (29.2)	30 (30.0)
Residence		
Urban	111 (92.5)	77 (77.0)
Rural	9 (7.5)	23 (23.0)
Education status		
Illiterate	28 (23.3)	17 (17.0)
Semiliterate^a^	36 (30.0)	26 (26.0)
Secondary	28 (23.3)	27 (27.0)
University and above	28 (23.3)	30 (30.0)
Occupation		
Manual worker	9 (7.5)	11 (11.0)
Public service employee	20 (16.7)	24 (24.0)
Retired	24 (20.0)	18 (18.0)
Unemployed	60 (50.0)	40 (40.0)
Other^b^	7 (5.8)	7 (7.0)
Diarrhea		
No	84 (70.0)	99 (99.0)
Yes	36 (30.0)	1 (1.0)
CD4+ count (cells/μl)		
Mean ± SD	934 ± 124	3490 ± 2761
< 200	21 (17.5)	0 (0.0)
200–349	11 (9.2)	0 (0.0)
350–500	16 (13.3)	0 (0.0)
> 500	72 (60.0)	100 (100)

*HD* Hemodialysis, *SD* Standard deviation; ^a^ includes those barely able to read and write, ^b^ professionals and businessmen

### Comparison between intestinal parasitoses among HD patients and apparently healthy counterparts

The overall prevalence of intestinal parasitoses was significantly higher (*P* < 0.001) among HD patients compared to their apparently healthy counterparts (52.5% vs. 12.0%, respectively). Among HD patients, *Cryptosporidium* species (32.5%), *B. hominis* (24.2%) and microsporidia (11.7%) were the most frequent parasite species, while *E. coli, G. lamblia, C. belli* and *C. cayetanensis* (1.7% each) were the least frequent species. On the other hand, *B. hominis* (13.0%), *Cryptosporidium* species (11.0%) and *G. lamblia* (4.0%) were the most frequent parasite species among apparently healthy individuals*.*None of the helminthic parasites was detected among HD patients or apparently healthy individuals. Differences between the two groups were statistically significant for the infection rates with *B. hominis, Cryptosporidium*species and microsporidia. Regarding the types of infection, there were statistically significant differences between HD patient and apparently healthy individuals in single infections (31.7% vs. 3.0%, respectively) and double infections (13.3% vs. 2.05, respectively). However, there was no statistically significant difference between the HD patients and apparently healthy individuals in triple infections, being 7.5% vs. 7.0%, respectively (Table 2). On the other hand, no statistically significant association was found between individual parasite species and diarrhea among HD patients (Table 3).

**Table 2:** Prevalence of intestinal parasitoses among HD patients and apparently healthy individuals in Alexandria, Egypt (2014–2016)

Parasite species	HD patients (*N* = 120)	Apparently healthy individuals (*N* = 100)	*P* value
	*n* (%)	*n* (%)	
Overall infection rate	**63** (**52.5**)	**12** (**12.0**)	< 0.001*
*E. histolytica/dispar*	4 (3.3)	2 (2.0)	0.691
*E. coli*	2 (1.7)	3 (3.0)	0.661
*G. lamblia*	2 (1.7)	4 (4.0)	0.414
*B. hominis*	29 (24.2)	13 (13.0)	0.036*
*Cryptosporidium* species	39 (32.5)	11 (11.0)	< 0.001*
*C. belli*	2 (1.7)	0 (0.0)	0.195
*C. cayetanensis*	2 (1.7)	0 (0.0)	0.195
Microsporidia	14 (11.7)	3 (3.0)	0.017*
Type of infection			
Single	38 (31.7)	3 (3.0)	< 0.001*
Double	16 (13.3)	2 (2.0)	0.002*
Triple	9 (7.5)	7 (7.0)	1.000

*HD* Hemodialysis; *, statistically significant at *P* < 0.05

**Table 3:** Association of parasite species with diarrhea among HD patients in Alexandria, Egypt (2014–2016)

Parasite species^a^	*N*	Presentation with diarrhea *n* (%)	*P* value
*Cryptosporidium* species			
Yes	39	12 (30.8)	0.530
No	81	24 (29.6)	
*B. hominis*			
Yes	29	8 (27.6)	0.469
No	91	28 (30.8)	
Microsporidia			
Yes	14	4 (28.6)	0.586
No	106	32 (30.2)	
*E. histolytica/dispar*			
Yes	4	2 (50.0)	0.384
No	116	34 (29.3)	

^a^ Parasite species detected in diarrheic cases were only included; *N* Number examined; *n* Number presented with diarrhea

### *T. gondii* seroprevalence among HD patients and apparently healthy counterparts

Table 4 shows that the overall *T. gondii* seroprevalence was significantly higher (*P* < 0.001) among HD patients compared to apparently healthy counterparts (33.3% vs. 8%, respectively). Anti-*Toxoplasma* IgM antibodies were detected in four cases of HD patients (two alone and two with IgG) and in two cases of apparently healthy individuals (one alone and one with IgG). However, a significantly higher (*P* < 0.001) IgG seroprevalence rate was found among HD patients compared to apparently healthy individuals (31.0% vs. 7.0%, respectively).

**Table 4:** Seroprevalence of *T. gondii* among HD patients and apparently healthy counterparts in Alexandria, Egypt (2014–2016)

*T. gondii* seropositivity	HD patients (*N* = 120)	Apparently healthy individuals (*N* = 100)	*P* value
	*n* (%)	*n* (%)	
Overall	**40** (**33.3**)	**8** (**8.0**)	< 0.001*
IgM	4 (3.3)	2 (2.0)	1.000
IgG	38 (31.7)	7 (7.0)	< 0.001*

*HD* Hemodialysis; *, statistically significant at *P* < 0.05

### Prevalence of intestinal parasitoses and *T*. *gondii* among HD patients in relation to CD4+ counts

Table 5 shows that HD patients with CD4+ counts < 200 cells/μl were twice more exposed to intestinal parasitoses compared to those with CD4+ counts ≥ 200 cells/μl, but the difference was not statistically significant (OR = 2.041; 95% CI = 0.759–5.489, *P* = 0.158). However, low CD4+ count was significantly associated with a higher infection rate with *Cryptosporidium* species (52.4% vs. 28.3%), where patients with CD4+ counts < 200 cells/μl were at about 2.8-fold higher risk of infection than those with CD4+ counts ≥200 cells/μl (OR = 2.79; 95% CI = 1.066–7.297, *P* = 0.037). In addition, low CD4+ count was significantly associated with a higher infection rate with microsporidia (28.6% vs. 8.1%), where patients with counts < 200 cells/μl were at about 4.5-fold higher risk of infection than those with counts ≥200 cells/μl (OR = 4.55; 95% CI = 1.066–7.297, *P* = 0.037). On the other hand, Table 4 shows that low CD4+ count was significantly associated with a higher *T. gondii* seropositivity rate (61.9% vs. 27.3%), where patients with counts < 200 cells/μl were at more than fourfold higher risk of infection than those with counts ≥200 cells/μl (OR = 4.33; 95% CI = 1.617–11.610, *P* = 0.004).

**Table 5:** Association of CD4+ counts with intestinal parasitoses and *T. gondii* infection among HD patients in Alexandria, Egypt (2014–2016)

Parasite species	CD4+ count (/μl)	*N*	*n* (%)	OR (95% CI)	*P* value
Overall intestinal parasitoses	< 200	21	14 (66.7)	2.04 (0.759–5.489)	0.158
	≥ 200	99	49 (49.5)		
*Cryptosporidium* species	< 200	21	11 (52.4)	2.79 (1.066–7.297)	0.037*
	≥ 200	99	28 (28.3)		
*B. hominis*	< 200	21	6 (28.6)	1.32 (0.460–3.798)	0.604
	≥ 200	99	23 (23.2)		
Microsporidia	< 200	21	6 (28.6)	4.55 (1.383–14.970)	0.013*
	≥ 200	99	8 (8.1)		
*E. histolytica/dispar*	< 200	21	2 (9.5)	5.11 (0.677–38.510)	0.114
	≥ 200	99	2 (2.0)		
*G. lamblia*	< 200	21	0 (0.0)	0.907 (0.042–19.580)	0.950
	≥ 200	99	2 (2.0)		
*C. cayetanensis*	< 200	21	1 (4.8)	4.90 (0.294–81.660)	0.268
	≥ 200	99	1 (1.0)		
*C. belli*	< 200	21	1 (4.8)	4.90 (0.294–81.660)	0.268
	≥ 200	99	1 (1.0)		
Overall *T. gondii* seropositivity	< 200	21	13 (61.9)	4.33 (1.617–11.61)	0.004*
	≥ 200	99	27 (27.3)		

*HD* Hemodialysis, *OR* Odds ratio, *CI* Confidence interval; *, statistically significant at *P* < 0.05

## Discussion

Opportunistic parasitic infections are a major cause of morbidity and mortality in immunocompromized patients, particularly those with low CD4+ counts [11–13]. Increased blood urea levels in patients with ESRD could lead to a weakened immune system and increased risk of morbidity and mortality associated with such opportunistic infections [30]. On the other hand, HD patients are more prone to such opportunistic infections, where repeated HD decreases CD4+ counts compared to predialysis and healthy controls [31]. Up to the best of our knowledge, the present study is the first to compare infection rates with intestinal parasites and *T. gondii* between HD patients and apparently healthy individuals in relation to CD4+ counts in Alexandria, Egypt.

The overall prevalence rate of intestinal parasitoses among HD patients was significantly higher than their apparently healthy counterparts (52.5% vs. 12.0%, respectively). Lower infection rates among HD patients were reported from Brazil (45.1%), Turkey (43.7%) and Iran (11.9–30.7%) [13, 32–34]. Compared to the finding of the present study, higher infection rates with *Cryptosporidium* species (40.0%), *E. histolytica* (14.0%) and *G. lamblia* (12.0%) have been recently reported among CRF patients undergoing HD in Upper Egypt [16]. The differences in the prevalence rates of infections between HD patients in the present study (north of Egypt) and those in the latter study (south of Egypt) could be attributed, among others, to differences in environmental, sanitary and hygienic factors. However, the reasons for such geographical differences in the prevalence of parasites among HD patients require further investigations.

In line with the findings of the present study, *Cryptosporidium*species (26.4%) and *B. hominis* (24.5%) were the most prevalent intestinal parasites among Brazilian HD patients [12]. Among the controls, however, *B. hominis* was the most prevalent species while *Cryptosporidium*species was not detected [12]. Another Brazilian study also reported *B. hominis* as the most prevalent parasite species among HD patients (20.1%) followed by *Endolimax nana*(16.3%), while *Cryptosporidium* species was reported among 4.7% of patients [31]. Similarly, *B. hominis*(4.2–14.1%) and *Cryptosporidium* species (11.5%) were the most common parasite species among Iranian HD patients [13, 34–36]. In addition, *B. hominis* (23.9%) followed by *G. lamblia* (8.5%) were the most prevalent parasite species among Turkish ESRF patients undergoing HD, while low rates of *Cryptosporidium* species, *E. histolytica* and microsporidia (2.1% each) were detected among patients but not the controls [33]. The variations in the prevalence of intestinal parasitoses among HD patients could be partly explained by the differences in the geographical distribution of parasites at the community level as a result of environmental, climatic and sanitary differences in addition to differences in hygienic and behavioral factors at the individual level. In addition, the role of the immune status and duration of HD as well as the method of stool examination in infection rate differences could not be ruled out.

In the present study, approximately a third of HD patients infected with the opportunistic parasites (*Cryptosporidium* species, *B. hominis* or microsporidia) had diarrhea, even though with no statistically significant association. Although the role of *B. hominis* in human disease is controversial [37], it can be a cause of diarrhea in patients on regular HD and renal transplant recipients [38, 39]. The lack of a statistically significant association between individual parasite species and presentation with diarrhea among HD patients in the present study is in contrast to the recent finding among CRF patients undergoing HD in Upper Egypt, where a statistical significance was found between *Cryptosporidium* species and diarrhea [16]. It also disagrees with that reported among Saudi patients undergoing HD [40], where *Cryptosporidium* species, microsporidia and *G. lamblia* were significantly higher among diarrheic patients compared to diarrheic controls. However, it is in agreement with that reported among Brazilian HD patients, where no association was found among individual parasite species and diarrhea [32]. In fact, the lack or presence association of HD with diarrhea among patients from different countries could be explained by differences in the level of immunosuppression, which determines the presence and severity of diarrhea. It is noteworthy that the differences in the incidence of gastrointestinal symptoms, including diarrhea, among CRF patients undergoing dialysis could be largely attributed to a number of factors such as the levels of uremic toxins, presence of metabolic co-morbidities, intake of medications and psychosocial factors [41, 42]. On the other hand, absence of helminths among HD patients in the present study is consistent with an earlier study among immunocompromized patients from Alexandria [43].

The overall *T. gondii* seroprevalence rate among approximately a third of HD patients in the present study is lower than those previously reported among HD patients (61.7%) and renal transplant recipients (70.0%) in Alexandria [17]. Similarly, it is lower than the rates reported from Turkey (56.0–76.5%) [44, 45], Mexico (56.7%) [46] and Iran (56.7–73.7%) [47–49]. The anti-*Toxoplasma* IgM seroprevalence among HD patients in the present study (3.3%) is lower than those reported among Egyptian patients on regular HD (16.7%) and renal transplant recipients (24.1%) [50]. In addition, it is lower than those (7.8–13.5%) reported among Iranian patients undergoing regular HD [49, 51]. In general, the seroprevalence rate of anti-*Toxoplasma* IgM among HD patients in the present study is somewhat close to the rates reported from Turkey (1.7%) and Iran (2.0%) [44, 47]. However, comparisons can be difficult due to a number of factors such as the duration of HD and the severity of renal damage. Because of the difficulty in adopting molecular techniques for the diagnosis of *T. gondii* infection in resource-limited countries, serological detection of anti-*Toxoplasma* antibodies is still the most commonly used approach for the screening of the infection among immunocompromized patients [52]. However, a major limitation of the present study is the inability to confirm whether the three cases seropositive for both IgM and IgG were acute infections because IgM may persist for years [53, 54]. A good approach to the real-time serodiagnosis of *T. gondii* infection that distinguishes between acute and chronic infections is the measurement of IgG avidity [55]. In this regard, the low avidity of specific anti-*Toxoplasma* IgG indicates a recent primary infection [56]. In addition, development of kits using immunoreactive proteins and multi-epitope antigens has been suggested for improving the diagnosis of *T. gondii* infection [57–59]. The high *T. gondii*seronegativity rate among HD patients in the present study indicates that the majority of HD patients had not been exposed to infection and are susceptible to the risk of acute infection.

In the present study, 40.0% of HD patients had CD4+ counts < 200 cells/μl compared to > 500 cells/μl in all apparently healthy individuals. It is noteworthy that CD4+ count of < 200/μl is categorized as a severe level of immunosuppression [29]. Such low CD4+ counts among HD patients were significantly associated with the infection with opportunistic parasites *Cryptosporidium* species, *B. hominis*, microscopridia and *T. gondii*. The significant association between low CD4+ count and infection with *Cryptosporidium* species in the present study is consistent with that reported from Saudi Arabia, where a significantly higher rate of infection with *Cryptosporidium*species was found among HD patients with CD4+ counts of < 500 cells/μl compared to those with CD4+ counts of > 500 cells/μl (66.7% vs. 29.6%) [39]. In another context, CD4+ counts of < 200 cells/μl were significantly associated with infection with *Cryptosporidium* species, *C. belli* and microsporidia among HIV-infected patients on antiretroviral therapy from Cameroon [60]. On the other hand, the significant association between low CD4+ count and *T. gondii* seropositivity in the present study is in line with that reported among Indian HIV-infected patients [61, 62], where a significant difference was found between the mean CD4+ counts in *Toxoplasma*-seropositive and -seronegative patients and that most IgG-seropositive patients had CD4+ counts of < 100/μl. Similarly, CD4+ counts of ≤100/μl were also found to be significantly associated with IgG seropositivity among HIV-positive patients without neurological complication from Nigeria [63].

One of the primary limitations of the present study comes from its observational design and that the prevalence of intestinal parasitoses was determined at a single point in time. The temporal relationship of the duration of HD with CD4+ counts and, hence, prevalence of parasites was not studied in the present study. However, this study reveals an absence of helminthic parasites that are usually detected by routine stool examinations as well as a high prevalence of *Cryptosporidium* species, *B. hominis* and microsporidia that are only detected after staining with specialized stains. This, in turn, necessitates the importance of considering the special request for such staining procedures by physicians as part of the routine diagnostic tests for HD patients. Another limitation is the inability to confirm serodiagnosis because no IgG avidity testing was done and single serum samples were tested.

## Conclusions

Intestinal parasitoses and *T. gondii* infection rates are significantly higher among Egyptian HD patients compared to apparently healthy individuals. Moreover, *Cryptosporidium* species, *B. hominis*, microsporidia and *T. gondii* are the most frequently detected opportunistic parasites among HD patients. The significantly higher past exposure to *T. gondii* infection among HD patients compared to their counterparts can pose these patients to the risk of infection reactivation because of their immunocompromized state. In addition, the high rate of *T. gondii*seronegativity can threaten their lives as a result of the possibility of exposure to infection during HD. CD4+ cell counts < 200 cells/μl can be significantly associated with a higher infection rate with *Cryptosporidium* species, microsporidia and *T. gondii* among Egyptian HD patients. Therefore, regular screening of HD patients for opportunistic intestinal parasites using specialized staining techniques and serological testing for *T. gondii* are recommended as part of the overall healthcare of such patients. Further large-scale studies on parasitic infections and associated risk factors among HD patients and other categories of immunocompromized patients are recommended.

## Data Availability

Data of this study are available from the corresponding author upon reasonable request.

## Abbreviations

CD4+: Cluster of differentiation 4
CI: Confidence interval
CRF: Chronic renal failure
EDTA: Ethylenediaminetetraacetic acid
ESRF: End-stage renal failure
HD: Hemodialysis
HIV: Human immunodeficiency virus
IgG: Immunoglobulin G
IgM: Immunoglobulin M
OR: Odds ratio
SD: Standard deviation
SPSS: Statistical Packages for Social Sciences
